# “The English Disease” or “Asian Rickets”? Medical Responses to Postcolonial Immigration

**DOI:** 10.1353/bhm.2007.0062

**Published:** 2007

**Authors:** Roberta Bivins

**Keywords:** assimilation, British Asian, ethnicity, immigration, osteomalacia, postcolonial medicine, rickets

## Abstract

Do the former colonizing powers, like their former colonies, have “postcolonial medicine,” and if so, where does it take place, who practices it, and upon whom? How has British medicine in particular responded to the huge cultural shifts represented by the rise of the New Commonwealth and associated postcolonial immigration? I address these questions through a case study of the medical and political responses to vitamin D deficiency among Britain’s South Asian communities since the 1960s. My research suggests that in these contexts, diet frequently became a proxy or shorthand for culture (and religion, and race), while disease justified pressure to assimilate.

As historians have demonstrated over the last two decades, the practice of medicine and medical research across a diversity of colonial and imperial contexts shared a particular relationship with power, whether that power be examined at the institutional, the political, or the social level.^1^ Medicine sought, and in policymaking circles came to have, a normative voice—one that resonated through the colonized world, pervading the processes of observation and interpretation and the evaluation of cultural, as well as biological, phenomena. This authoritative voice spoke from medical and scientific institutions embedded in the metropolitan centers of empire, and responsively or reiteratively from research outposts and field stations on empire’s perceived “periphery.” Of course such a “call and response” relationship was only ever an imperial ideal: colonial populations, and ambitious (or culturally acute) researchers and practitioners in both locations, resisted their roles.^2^ But both the ideal and the empires were powerful, and in London, Paris, and Washington the fiction (at least) of a discrete center managing and studying a far-flung periphery was easily maintained well into the twentieth century. So what happened when the empires disappeared, and colonial subjects themselves colonized the metropole?

Historians have recently begun to examine postwar medicine in the new nations that replaced Europe’s imperial colonies under the rubric “postcolonial medicine.” Research approaches (and thus outcomes) in the history of medicine have followed two major paths: some scholars have used the term “postcolonial” largely empirically, to designate the[Other P-534] period from the decolonization of an individual region or state to the present, and events within that period. Others have incorporated into the term a number of theoretical models, particularly implicit or explicit comparisons with the colonial state, its institutions, and its zeitgeist. Those following this latter path have focused on issues relating to orientalism or the creation of “subaltern” groups; the role of nationalism and national identities; the “indigenization” of scientific, medical, and educational institutions and bureaucracies; and consequent changes (or stabilities) in attitudes toward indigenous medicine, race, and cultural practices. Unsurprisingly, scholars in this mode do not confine their investigations to the period after formal decolonization, but rather incorporate that process and its more or less immediate antecedents within their remit. Both approaches have tended to focus on individual nations (while, often implicitly, assuming that general trends exist). Few of either school have closely interrogated the impact of decolonization on medicine in the once-colonizing powers.

Here, I certainly use the term “postcolonial” to indicate the period from the end of World War II until the present; my investigations will not delve extensively into colonial matters. However, I also incorporate the connotation that the social and cultural effects of colonialism do not simply disappear with its political and governmental apparatus, and that therefore researchers should interrogate emergent practices and institutions in the light of their colonial predecessors and antecedents. If, as much current scholarship in the history of medicine suggests, “colonial medicine” was transformative of medical practice and medical knowledge in colonizing, as well as colonized, societies, then its impact should persist and shape medicine, broadly construed, in the former as well as the latter locales, even after the end of formal empire. Thus I ask, did a distinctively “postcolonial” form of medicine emerge in the former imperial centers, as it did in the former colonies? If a metropolitan “postcolonial medicine” does exist, what does it look like, who practices it, and upon whom? Using the case of rickets and osteomalacia in Britain’s diverse South Asian communities since the 1950s, I will argue that the bodies of immigrants and ethnic minorities became the metropolitan sites of imperial medicine’s “postcolonial” development.

Britain struggled with terrible labor shortages in the years during and immediately following World War II. It addressed this problem in part through a very active program of labor recruitment in its dominions, colonies, and former colonies, building on the long-held imperial tradition that all British subjects were entitled to unrestricted entry to the United Kingdom: it was, after all, “home.” As other historians have noted, this[Other P-535] “Windrush generation”—though numerically relatively small—produced a very visible impact on the cultural and racial make-up of many British towns and cities in the 1950s and 1960s.^3^ In turn, this sparked (or further inflamed) significant social and political tensions, particularly over shortages in public housing, levels of crime, and costs and access to Britain’s welfare state (including the National Health Service).

As the postwar economy and reconstruction work slowed in the 1960s, Britain’s appetite for foreign (and especially unskilled) labor diminished. A sense of a shrinking labor marketplace, as well as anxieties about racial and cultural mixing, fueled demands for controls on immigration and the dispersal of immigrant and ethnic communities. Outbreaks of violence in highly diverse communities further exacerbated indigenous fears and, some scholars argue, politicized immigrant and ethnic populations as well.^4^ As in the United States, issues of medicine and public health were intimately involved in the immigration debates.^5^

This, then, was the context in which British medical practitioners and policymakers found themselves again facing an “old” illness. Rickets in the postwar era has been widely regarded by doctors and scholars alike as a “historical” disease—a disappearing symptom of Victorian industrialization and urbanization. Reconsidering it through a postcolonial lens encourages us to drag our gaze away from this nineteenth-century disease experience (and the expectations with which that weight of history has imbued rickets) and to refocus on the condition as potentially “foreign,”[Other P-536] “rare,” and “racial”—and thus as an effective model of the complex medical environments and communities of mid-to-late twentieth-century Britain. Both the visible medical response to postwar rickets and the internal debates of central governmental departments suggest that for medical researchers, policymakers, and politicians, immigrants’ habits of diet and dress could become proxies or shorthand for culture (and religion, and race).^6^ Rickets and osteomalacia, precisely because of their tractability to both behavioral choices and biomedical interventions, were used to justify pressure to assimilate—and conversely, if less commonly, to highlight social and medical inequities.

At the same time, the case studies of elite research units working with rickets in Manchester and London also illustrate certain continuities between colonial and postcolonial medicine, not least in the ways in which the newly available postcolonial bodies (like those of their colonial predecessors) were co-opted by existing research programs and interpreted through the lens of ongoing debates within communities of clinicians and medical researchers.^7^ Through a brief comparison with one colonial study of the same diseases, I suggest that patterns of study of rickets—particularly its adoption as a topic of interest by biochemists and geneticists—reflect patterns established by metropolitan researchers of tropical diseases in the colonial era. Thus, elite researchers in the postcolonial metropolis, like their predecessors in the colonial one, focused closely on the scientific problems presented by the complex biochemistry of the disease rather than on more mundane questions of epidemiology or the development of effective (culturally sensitive) strategies of prevention and treatment.^8^ Those questions were left to nutritionists, public health workers, and clinicians in areas of heavy immigration—areas newly colonized [Other P-537] by the postcolonial immigrants.^9^ Gender (and ethnicity) too played an important role in this division of medical labor, in both the colonial and the postcolonial contexts.

## Nutritional Research and Tropical Medicine: Rickets and Osteomalacia in Colonial India

In 1929 Walter Fletcher, secretary of the British Medical Research Council (MRC), returned a set of research reports to George Newman, chief medical officer to Britain’s Ministry of Health (MoH).^10^ The reports detailed the findings of two female medical researchers, Dr. Dagmar Curgel Wilson and Professor Ella Surie, on osteomalacia (or “late rickets”) among [Other P-538] women and girls in northern India. In ways ranging from the intraprofessional to the sociopolitical, their reports neatly exemplify “colonial medicine,” particularly in relation to gender. It is likely that Wilson (before her marriage a member of India’s Women’s Medical Service) and Surie (then professor of physiology at Delhi’s Lady Hardinge Medical College) would have struggled to find posts and research funding in the interwar metropolis; in India, however, their gender was the key to unlocking the *zenanas* (enclosed women’s quarters) and rendering available for study the bodies of women and girls. Thus Western female researchers, like the pioneering women doctors before them, were encouraged to go to India “where they were needed,” while Indian women were admitted, if not actively recruited, into the growing indigenous scientific workforce.^11^ Indeed, Fletcher had made both the need for female researchers and his agenda for their research explicit in an earlier letter: “As to osteomalacia in India, I have tried hard to stir up interest among various women medical workers there in the new knowledge we have relating ossification to diet, and I think there is some hope of work being done.”^12^ And while Wilson and Surie took care to represent themselves as members of an international research community, they also portrayed their work as largely empirical and thus, to an extent, subsidiary and responsive to the analytical agendas of researchers in the metropolitan centers. Unsurprisingly, they were particularly attuned to the MRC’s drive, guided by Fletcher—himself a biochemist by training and a champion of biomedical research—to promote dietary factors as the principal cause of deficiency rickets/osteomalacia, and to move away from hypotheses rooted in older models of “geographical or social difference.”^13^ Finally, as was typical of the doctor-patient relationship in late colonial medicine, the women and girls who were the objects of Wilson’s study were questioned, observed, examined, measured, categorized—but neither individually identified nor ascribed by researchers with any meaningful agency in their dietary or behavioral choices. Moreover, their self-reporting, although necessary, was discounted, and the data derived from it were subject to considerable qualification: “It is extremely difficult to obtain reliable evidence with regards to the amount of sun obtained, and in some cases where the [Other P-539] housing conditions of the patient have been known to be bad, the considerable amount of sun said to be obtained, has been discredited.”^14^

And yet studies of nutrition and diet, wherever they were performed, could not adopt wholesale or exclusively the laboratory-driven model of “objective” biomedical research, however attractive from an imperial point of view. The researchers depended heavily on data about daily life readily available only to its objects—in this case, the Indian girls and women themselves—and were necessarily interested in customs, practices, and identities. In this discipline, even metropolitan studies of nutrition and public health were forced to adopt colonial medicine’s nearly anthropological methods. And as the case of rickets and osteomalacia in postwar Britain will demonstrate, when those metropolitan studies were also addressing non-white bodies, they often echoed the focus on “racial habits”^15^ that had been a consistent and (at least by the interwar period) largely distinctive feature of biomedicine as practiced in the colonies and on colonial bodies.^16^

Fletcher’s response to Wilson and Surie’s studies, and to nutritional-deficit diseases in India generally, also typifies the colonial mode and moment. His reply to Newman enthused: “there is a great field for work in India by trained investigators and especially with regard to nutrition as related to osteomalacia and rickets. . . . Any progress they make will not only help India enormously, *but will help us by suggesting new problems for more primary work by investigators here*.”^17^

In such discussions of medical research in India, Fletcher and his colleagues were treading a very familiar path. It was characteristic for empirical work in the colonies to be regarded—at least by researchers within elite Western institutions—as providing raw material for analytical work in the metropole. Similarly, colonially based medical research commonly pathologized social practices of which the colonial power and colonizing culture disapproved.^18^ Thus, for example, Wilson and Surie commented [Other P-540] extensively and critically on the Islamic religious practice of enclosing women (a practice widely condemned as “uncivilized” by Europeans), justifying their criticisms on medical grounds. Simultaneously, they took advantage of the specific results of this cultural difference to further their research agenda: “This practice of purdah is deeply to be deplored, since the greater severity of the disease among this particular group of the community is direct evidence of the need for ample sunlight to compensate dietic [*sic*] deficiencies.”^19^ Thus clinical research and observations in the colonies provided both material for scientific progress—“more primary work”—in the metropolitan centers, and matériel for the social and political battle against “primitive” or “uncivilized” cultural practices in the colonies themselves.

Wilson and Surie’s observations—as was often the case for colonial research work—reached a relatively small, if powerful, audience.^20^ Not until the late 1950s would British medical attention be focused closely on rickets and osteomalacia in South Asian bodies—and by then, the “tropical” bodies had become very local. Instead, British research in this period looked inward: into the signs and symptoms of an increasingly subtle disease; into the metabolic machinery that underpinned those signs and symptoms; and into the diets of Britain’s own urban indigenes.^21^

## Rickets and Osteomalacia in Britain: Diet and Metabolic Research, 1900–1960s

Rickets and, in adolescents and adults, osteomalacia are conditions caused by vitamin D deficiency: without vitamin D (actually a steroid hormone rather than a vitamin in the modern sense), the body cannot absorb calcium or phosphorus, and therefore cannot properly build bones. In the [Other P-541] nineteenth and early twentieth centuries, rickets flourished in Northern Europe and North America, particularly among the urban (often immigrant) poor. Although the mechanism by which vitamin D is produced and functions in the body remained only partially understood until the 1980s and 1990s, researchers knew by 1918 that the vitamin D deficiency diseases could be cured by feeding cod-liver oil and, by the early 1920s, that they could also be treated by direct UV radiation and the radiation of certain foodstuffs. Vitamin D became known as the “sunshine vitamin.”^22^ The relative importance of environmental and nutritional factors in the generation of rickets, however, remained hotly debated, illustrating continued polarities between clinical and laboratory models of medical research.^23^

With improved air quality, housing, and diet, as well as new understandings of supplementation, florid nutritional rickets had virtually disappeared from British cities by the 1930s, somewhat to the disgruntlement of clinicians involved in medical education, particularly in pediatrics.^24^ Clinical attention to vitamin D deficiency rickets in the following decade was devoted largely to the problem of identifying the now-curable disease as early as possible—so early, in fact, that no consensus could be reached on clinical diagnosis, or on the degree of continued prevalence of the condition. Debates initially focused on the contest between tactus eruditus (and the craniotabes that only it could uncover) and the X ray, and gave birth to the category “radiological rickets.”^25^ The shrinking literature of this period acknowledged the poverty and nutritionally inadequate diet that were the shared background of all rickety babies, generally without stigmatizing the families involved. The *Lancet*, for example, editorialized in 1940 that

it must be galling to the research workers, laboratory and clinical, who have given the whole story of rickets a reasonably simple explanation in terms of vitamin-D deficiency to find [that] “mild cases are still seen too frequently even [Other P-542] amongst infants attending welfare clinics” . . . it is reasonable to suspect that economics rather than lack of the application of scientific knowledge explains the present-day occurrence of mild rickets.^26^

Vitamin D deficiency was, of course, a medical concern during World War II, as the Medical Research Council actively campaigned for the enrichment of basic foodstuffs. It is far from coincidental that the secretary of the MRC in this period was Edward Mellanby, who first defined rickets as a nutritional-deficiency disease (initially disregarding environmental factors entirely) and one that could be prevented by dietary supplementation.^27^ From the outbreak of war until the end of rationing in 1954, the British government actively pursued a three-pronged strategy of intervention into the nutrition of its population: it used regulation to enforce the fortification of flour with calcium, and of margarine with vitamins D and A, and the exclusive milling of bulky, nutrient-rich (but brown and unprofitable), high-extraction flour. It controlled the national diet via rationing and the provision of special diets and supplements to particular groups (for example, expectant and nursing mothers, infants, young children, and hospital patients). And finally, wartime governments avidly supported nutritional education, woven into wider propaganda programs.^28^

After the war, small- or large-dose supplementation continued to be the routine clinical response to nutritional rickets, although “careless” or “preoccupied” mothers were increasingly blamed for their children’s now-rare condition.^29^ And central governments likewise continued to make such supplements (in the form of cod-liver oil) available for free, or at heavily subsidized rates, for families with young children already taking welfare foods or with a doctor’s order.^30^ The flow of clinical papers on nutritional rickets became a trickle, and it ceased to be a matter debated on public-policy grounds. The literature on the biochemistry of vitamin D metabolism, meanwhile, expanded significantly. The extraordinary decline in [Other P-543] simple vitamin D deficiency rickets had revealed a spectrum of different rickety syndromes: rickets caused by metabolic disorders rather than nutritional inadequacy. Through studying these complex cases, advances could be made in the prestigious (and, with academic researchers establishing ever-closer collaborations with the pharmaceutical industry, potentially lucrative) research fields of biochemistry and genetics.^31^

Simultaneously, studies and criticisms of mass supplementation through the fortification of basic foodstuffs increased, particularly as the era of rationing drew to a close. From 1951 to 1964, successive Conservative governments—and more determinedly, the Treasury bureaucracy—sought to free themselves (and food manufacturers) from the obligation of managing the British diet through the provision of welfare foods and mandatory fortification.^32^ In the case of vitamin D fortification, a complicating factor added urgency to the changing trend of medical policy: the emergence of a new clinical entity—hypercalcemia. This condition was characterized by an excess of calcium in the blood, leading to “failure to thrive” and, in severe cases, osteosclerosis and mental retardation among children consuming high levels of fortified foods, or fortified foods plus proprietary supplements.

By 1955, the Ministry of Health requested the British Paediatric Association “to enquire into the incidence of infantile hypercalcaemia and to consider whether the disease had any relationship to the intake of vitamin D.”^33^ In 1956, the Medical Research Council sponsored a conference on the same topic; the conference report concluded that

more cases [of hypercalcaemia] have been reported in Great Britain and Northern Ireland than in other parts of the world. There is evidence that the fortification of foods has materially increased in Great Britain. . . . The disease does, however, appear to be rare in the United States where fortification is less extensive, although the practice of supplementation is probably more common.^34^

In fact, subsequent studies revealed that in the period 1953–55 approximately a dozen cases in total were reported in the United States, while the United Kingdom averaged almost a hundred per year.^35^ As a new and apparently iatrogenic disease, hypercalcemia forced the relevant ministries seriously and swiftly (at least by ministry standards) to reconsider the wartime levels of vitamin D supplementation, which had eradicated rickets from the indigenous population.^36^ The MRC report had concluded that “it is only possible to recommend a range of intake which will be sufficient to protect all but the most resistant children against rickets and at the same time only expose the most responsive children to the risks of hypercalcaemia”;^37^ they suggested a total of 400 i.u. of vitamin D from all sources—a figure that necessitated some reduction in levels of fortification, and especially in the fortification of infant cereals. By 1957, the Ministry of Health changed its recommendations for fortification and supplementation in line with both its own and the MRC’s findings; their goal was to protect the vast majority of British children from hypercalcemia, without enabling a return of rickets to that population. This commonsense approach would come to have unexpectedly serious implications for the health of ethnic minority populations, and in turn for the health of race relations in the medical sphere. It meshed perfectly, however, with the broad trend of governmental policy to withdraw from regulation and control in the area of nutrition, and to intervene in the national diet via health education alone. Governments still encouraged manufacturers to fortify various dietary staples to recommended levels; however, with the exception of infant-formula producers, millers (still compelled to fortify white flour), and margarine producers (likewise still required to fortify their product with vitamins A and D), manufacturers experienced no compulsion.^38^

Partly as a result of reductions in fortification, partly as a result of increasingly biochemical understandings of the D deficiency diseases, and, as we will see, partly because of the emergence of a new “at-risk” population, rickets again became the focus of considerable clinical and media attention. It reentered the policy arena in the late 1950s and 1960s. The decline of overt rickets, and the intense biochemical investigation of blood chemistry in the 1950s and early 1960s, had brought with them yet another contested category of rickets: “biochemical rickets.” Initially diagnosed by blood tests for abnormally low levels of serum calcium and raised levels of serum alkaline-phosphatase, “biochemical rickets” could be discovered in children and adults showing neither clinical nor radiological signs of disease. Obviously, such a definition of rickets/osteomalacia greatly increased its rate of incidence (particularly among the majority community), and therefore the public health significance of the condition—at least for those who accepted the category. Not everyone did: policymakers and the ministries resisted the idea of asymptomatic rickets well into the 1970s. In response to a 1973 parliamentary question from Baroness Summerskill, for example, Lord Aberdare told the House of Lords rather dismissively: “Recent reports of . . . alleged ‘biochemical rickets’ that is, without clinical signs, in the indigenous population of one city are being considered by an expert panel of the Committee on the Medical Aspects of Food Policy and it is too early to say what, if any, action is needed.”^39^

As Aberdare’s response suggests, nutritional rickets and osteomalacia, whether biochemical, radiological, or clinical, were still marked out as diseases of impoverished city-dwellers. As in the prewar period, such populations contained a significant proportion of immigrants—but this time, the affected immigrants were almost exclusively from the “New Commonwealth,” especially India, Bangladesh, and Pakistan. Whether recruited to ease Britain’s postwar labor crunch, driven out by poverty or the xenophobic regimes of new East African nations, or simply drawn by historic ties and the rhetoric of Commonwealth, “these people” became a highly visible—and not always welcome—new presence in industrial towns and cities across the United Kingdom.

It is in relation to this new population that both the continuities and the disjunctures of “postcolonial” medicine become clearer. Compare “tropical medicine”—arguably the archetypal discipline of colonial medicine—with the spectrum of medical responses to postcolonial immigration and the emergence of nonwhite ethnic communities in postwar Britain. [Other P-546] Tropical medicine had produced clear and important impacts on medical and social debates at home (for example, in relation to the spread of bacteriological models of public health, and to notions of “race,” eugenic and otherwise).^40^ And it had functioned as an arm of state interests and imperialism in terms of serving and protecting European communities in the tropical colonies, and preserving a productive tropical workforce. However, it also had a distinct research program, with its own priorities and methodologies: first, promoting germ theory (and later the biochemical approach to nutritional diseases) through bacteriology, parasitology, disease ecology, and a new combination of largely metropolitan laboratory research and tropical/colonial field observations; and second, focusing a campaigning spotlight on particular diseases.^41^

Like tropical medicine, medical approaches to the postcolonial immigrants often—both institutionally and intellectually—served state and industrial interests, and engaged actively with social and policy debates. But unlike the “tropical diseases” of colonial medicine, the diseases of (or at least the diseases commonly identified with) the emergent postcolonial communities in Britain—for instance, rickets, tuberculosis, leprosy, sickle-cell anemia, and thalassemia—were not organized into a single medical specialty with its own institutions, programmatic aims, or distinctive and consistent methodologies. Rather, the ailments of immigrant and ethnic populations became selectively visible to medical researchers when those conditions justified or facilitated ongoing research programs. [Other P-547] Investigations of osteomalacia and rickets in Manchester and in London offer useful and contrasting case studies of this phenomenon in relation to the question of “postcolonial medicine.”

## Migrants and Metabolism, Manchester and London

As local campaigners and health workers have noted, rickets and osteomalacia were far from the biggest or most pressing medical problems faced by Asian immigrants and their British-born descendants; in the years 1955–80, clinical rickets and osteomalacia were diagnosed in only a few score Asian children, adolescents, and elderly per year, and all were easily and successfully treated.^42^ There were, of course, higher rates of “biochemical rickets,” with some researchers estimating incidences as high as 70 percent in the Asian population,^43^ but these cases were even more readily treated. Nevertheless, alongside tuberculosis, smallpox, and later thalassemia, vitamin D deficiency rickets/osteomalacia became, particularly in the medical press, an archetypal “Asian” disease. The *Lancet*, the *British Medical Journal*, *Proceedings of the Nutrition Society*, and other prominent journals used the term “Asian Rickets” throughout the period covered by this paper, and well into the 1980s, to the apparent satisfaction of their wide professional readership, and even in articles actively disputing theories of unusual Asian susceptibility.^44^ There is, of course, a certain irony to this categorization, since rickets was known throughout the nineteenth [Other P-548] century as “the English disease” in tribute to its remarkable prevalence in England’s gloomy and heavily polluted industrialized cities.

Given its relatively minor epidemiological impact, and its tractability to simple, cheap, and effective medical treatment, why did rickets draw so much medical and policy attention to an otherwise underserved population? In part, this was because its reappearance in the United Kingdom was an affront to public expectations and eroded political and medical achievements. In 1967, W. T. C. Berry, then secretary of the Committee on Medical Aspects of Food Policy (COMA, the chief fact-finding and advisory panel to the MoH on nutrition and fortification), testily declared to the Nutrition Society’s annual meeting: “Rickets excited great emotion, largely because it is thought that it is reappearing and this is a sign of social regression.”^45^ Berry clearly regarded this “emotion” as excessive, speaking dismissively of “the attention that has been given to the minutest signs of possible vitamin D deficiency,”^46^ but even he had to admit that a COMA panel studying the subject in 1965–66 had discovered “overt rickets” in “small pockets, either socioeconomic or racial, which might respond well to fairly simple modifications of our existing fortification system.”^47^ A member of the COMA Panel on Child Nutrition in 1972 was perhaps also responding to this sense of “social regression” when he described the reappearance of the disease as “rickets being introduced into the country.”^48^

“Asian rickets,” however, was not just an embarrassing public health problem: for some medical research communities, it was also a solution of sorts. Consider the case of metabolic medicine, and particularly the elite (and expensive) area of metabolic biochemistry. In 1963, the prestigious Manchester Royal Infirmary took the major step of opening a new Metabolic Unit, built with a £30,000 grant from the Wellcome Fund. S. W. Stanbury headed the Unit, and when the University made the additional significant commitment of endowing it as a professorial one in 1965, he was appointed as a second full-time professor of medicine. This joint institutional investment reflected the view that such disorders were part of “the new frontier” (encompassing the transition from acute/infectious to chronic/genetic disease, and from cellular to molecular understandings of disease processes) in medicine, and illustrates the status accorded to metabolic research.^49^

Studies of vitamin D metabolism and related aspects of lipid metabolism in both chronically ill and acutely ill patients were among the Metabolic Unit’s early topics of research, and thus at the heart of its institutional development and identity. But remember that the vitamin D deficiency diseases were in steep decline across the United Kingdom in this period. A 1960–61 survey performed by the British Paediatric Association (cajoled by the Ministry of Health), and intended to explore the impact of reductions in vitamin D fortification on the incidence of both rickets and hypercalcemia, showed extremely low rates of both conditions in the native population: “In 1960–61, the total of rickets (in non-immigrants) reported to the British Paediatric Association . . . by all paediatricians in Britain was 1.5 per month, and in 1961–62, 1.0 per month.”^50^ As their qualification “in non-immigrants” implies, this study was already suggesting a different picture for Britain’s immigrant population.

In London, Charles Dent, professor and principal investigator at the University College Hospital (UCH) Metabolic Ward, which he had opened in 1951, was also becoming aware of a shift. In 1960, he had responded eagerly when consulted on a suspected case of nutritional (or, in his preferred term, “classical”) rickets in an immigrant child: “The family being Turkish Cypriots makes me think very strongly that we may have an instance here of classical rickets due to oral dietary deficiency, undoubtedly the rarest cause of rickets nowadays in British people”; Dent mused: “curiously enough we see it much more often in visiting imigrants [*sic*] from backward countries.”^51^

Few cases of nutritional rickets were yet finding their way to specialist units.^52^ The Manchester and London researchers alike therefore initially studied British patients with “resistant rickets”: rickets resulting from congenital abnormalities, surgical interventions, or other pathological events, which had become visible only after nutritional rickets had been eliminated from the indigenous disease-picture. Although biochemically revealing, such cases were not only rare, but also, by definition, physiologically abnormal. Thus Dent’s enthusiasm for his Turkish Cypriot patient [Other P-550] was certainly related to a long-running study on the possible antirachitic action of a particular form of vitamin D (dihydrotachysterol, or DHT). Dent was desperate to find cases of “true rickets” on which to test the conclusions that he had reached in treating his metabolic patients (but which conflicted with animal-model studies performed twenty years before). As early as 1956, he had written to a colleague: “We have been treating metabolic forms of rickets for some time here with DHT and they do exceedingly well. . . . However, I am not completely happy about its use even in these cases in view of its alleged reputation for being unable to heal true rickets. I therefore began to organise a trial as opportunity arose.”^53^ He noted plaintively that thus far he had only been able to test two cases, both actually based in Dublin.

By contrast, from the mid-1960s, the rapidly growing British Asian communities of Greater Manchester and Central London offered an ample supply of rickets sufferers with normal metabolisms but unusual—and apparently rachitic—diets. In Manchester, by virtue of their high incidence rates alone, this section of the poor urban population suddenly found itself at the very center of scientific and experimental medicine as “clinical material.”^54^ The local ethnic community, with their cultural, dietary, and potentially hereditary differences, became a resource, rendering the Manchester Wellcome Metabolic Unit ideally positioned to study the prevalence and origins of vitamin D deficiency rickets: was it simply a question of poor diet, as had often been assumed in the colonial context?^55^ Of hereditary, or racial, maladaptation? Of unhealthy but traditional behaviors?

Of course, such questions had clinical and policy implications, of which Stanbury was well aware as a member of COMA. These were of singular importance to Medical Officers of Health, local health authorities, and the clinical staff of general hospitals in cities like Birmingham, Bradford, [Other P-551] and Glasgow, with large and expanding ethnic minority populations.^56^ (It may be worth noting at this point that the terms “Asian” and “British Asian” had to stretch to cover a wide diversity of distinct communities, with many different religious, dietary, and behavioral patterns. Although researchers and policymakers were ever more aware of these differences over the period from 1960 to 1980, initial public health responses focused primarily on linguistic difference: centrally prepared educational materials were conscientiously translated into a range of languages and distributed accordingly, and translators were sought. More-sensitive responses to increasingly sophisticated understandings of ethnicity came later, and initially from local, rather than national, initiatives.)^57^ But for the Metabolic Unit and other similar research groups, including the UCH Metabolic Ward and the Middlesex Hospital researchers discussed below, the solution to the puzzle of rickets in the population they generally called “immigrants” or “the Asian community” was of interest principally because it would contribute to unraveling the complex vitamin D cycle. We will see exactly this priority demonstrated in Stanbury’s response to a COMA initiative, aimed at addressing the resurgence of nutritional rickets.

In October 1972, COMA’s Panel on Child Nutrition, in response to heavy reporting in the biomedical press of vitamin D deficiency among British Asians and fears of its emergence in parts of the indigenous community, “concluded that expert working groups should be formed to deal with various aspects of the problem of rickets and osteomalacia in the immigrant population.”^58^ The remit of the working groups was to develop standardized methodologies for determining the incidence and severity of disease, and, if possible, its causes. COMA invited Stanbury to chair the clinical working group, alongside the pediatrician Professor C. E. Stroud of the Department of Child Health, King’s College Hospital, University of London. Two months later, the chairmen of the working groups met [Other P-552] to frame a plan of action. Stanbury opened discussions by noting that in the three preceding years he had seen “20–25 cases of overt rickets and osteomalacia admitted to hospital in the North of England. . . . In a survey of Asian immigrants in Rochdale [Greater Manchester], the incidence of overt rickets and osteomalacia was 30%; if abnormal biochemical results . . . were included in the definition, the incidence increased to over 70%.”^59^ This passage reveals, first, the numerical scarcity of cases, even within the ethnic communities with which they were most identified (by comparison, the previous decades’ moral panic over tuberculosis rates among immigrants involved hundreds of cases per year); and second, the profound impact that a researcher’s chosen definition of rickets made on the rates of disease incidence he or she reported.^60^

Stanbury and Stroud agreed to produce clinical questionnaires for administration to “at-risk” and “control” groups, assessing incidence of abnormality at all levels among each group. But as Stanbury later made clear in correspondence with Dr. Joan Stephen, secretary of the Panel on Child Nutrition and coordinator of the study, his commitment was first and foremost to the study of metabolic biochemistry, and any Manchester pilot study would have to advance that research:

The most important consideration is that we have a limited clinical staff in my Department and we are committed to a programme of research that leaves our resources fully stretched. At the same time, information of a biochemical nature that we might collect from examining particular populations could have relevance to our other work. . . . even if provided with additional clinical assistance, we would be reluctant to undertake further population work unless the effort returned information relevant to our main themes of research. *The point here is that we are primarily a biochemically orientated clinical research team. . . . further pilot examinations . . . could only be done if a by-product of our clinical examinations were the acquisition of biochemical information relevant to our personal research interests*.^61^

Stanbury laid out those biochemical interests in some detail; by contrast, his comments on the agenda of the Department of Health and Social Services (DHSS) seem an afterthought: “obviously it is fully appreciated that the primary purpose of any DHSS supported survey would be the compilation of a clinically appropriate questionnaire and system of physical [Other P-553] examination.”^62^ He immediately reverted to extolling the resources of the Manchester area as a base for primary research. Crucially, in this particular case, what made Manchester special was the availability “within minutes of this hospital” of large and diverse ethnic populations; Stanbury enthused: “local co-operation is so good that I have little if any doubt about [their] willing availability.”^63^

Compliance and cooperation were, of course, essential to metabolic researchers, perhaps even more than to clinicians and public health workers dealing with nutritional diseases on the ground. Stanbury found cooperative patients in the local South Asian communities served by—and geographically proximate to—the Metabolic Unit and the Manchester Royal Infirmary. But researchers also needed particular facilities to perform comprehensive studies: they needed a combination of highly specialized laboratory space and hospital beds, and not every metabolic research team was as fortunate as Manchester’s. The Middlesex Hospital’s new and technically sophisticated metabolic laboratory had no wards; a metabolic ward had long been based at University College Hospital, under Charles Dent, but had a strongly clinical focus and a full clinical research program of its own. By 1974, the two units were intent on formal links.^64^ In the meantime, the biochemically oriented Middlesex researchers, though operating in ethnically diverse central London, chose to seek their clinical material further afield, in Glasgow. For the Middlesex group, immigrant and ethnic bodies—and the political controversy stirred up by their poor nutritional status—provided the substrates (and perhaps also leverage with funding bodies) for innovative, even groundbreaking, research on the production and metabolism of vitamin D.^65^ And although entirely United Kingdom–based, their study almost perfectly replicated the colonial model of divisions—of both labor and locale—between laboratory and field research.

Even the earliest mentions of what would become a very well-known and controversial study of food fortification were intimately intertwined with basic research (funded, in accordance with their remit, by the MRC and the Wellcome Trust rather than the DHSS). The research group, headed by Middlesex’s Jeffery O’Riordan, based in an elite academic [Other P-554] Department of Medicine, were not primarily interested in rickets; rather, they developed interests in the role of vitamin D as a hormone in conjunction with studies of the pituitary and parathyroid hormones. In the course of their research into these systems, they had developed a highly sensitive assay that allowed them to assess “ambient vitamin D status” in individuals—necessary to determining “normal” levels of the hormone, and thus to recruiting appropriate control groups for a wide range of metabolic studies. The assay’s greater sensitivity was showcased in an initial, biochemical, study’s conclusions: “Until now, the serum-alkaline-phosphatase has been the most commonly used index of occult osteomalacia or rickets, but . . . it is clear that measurement of circulating 25-H.C.C. . . . will provide a more sensitive index.”^66^ The clinical and diagnostic advantages conferred by such a degree of sensitivity—necessary for the detailed study of a complex hormone system—were perhaps debatable, given existing controversies over “biochemical rickets”; but, like the Manchester team, the Middlesex workers were focused on basic research and the production of new biochemical, rather than clinical, knowledge. Indeed, at least in the eyes of one participant, the rickets and osteomalacia work at Middlesex was driven as much by the development of a newer and more precise method of assaying biochemical activity, and by the need for a research topic suited to the deployment of that new tool, as by the “hot” clinical problem.^67^

Nonetheless, the principal investigator’s subsequent bid for DHSS funding unsurprisingly stressed clinical applications and the public health “problem” posed by immigration. Writing informally to Dr. Stephen in her capacity as secretary to the Child Nutrition Panel and organizer of the working groups on rickets and osteomalacia in immigrants, O’Riordan was gung-ho both about his new tool, and about public health:

In our recent paper in *The Lancet*, we showed that in the serum of immigrants from India or Pakistan who have Rickets or Osteomalacia there is no detectable serum 25 hydroxy-cholecalciferol. This was studied using an assay for 25 hydroxy-cholecalciferol which we have developed and which I believe to be the most sensitive yet available. . . . You, I know, fully appreciate the magnitude of the problem posed by this group of the immigrant population. I, also, believe this to be a major public health problem, and it is one that I would like to attack [Other P-555] vigorously. . . . It seems to me likely that this is dietary in origin and that it is readily preventable by adding Vitamin D supplements to the diet. The problem is, of course, how best to get it into the diet of these immigrants.^68^

Like many medical professionals (the British Medical Association, for example, would call for prophylactic fortification at its annual representative meeting in 1977), O’Riordan saw fortification, rather than education, as the logical solution to the “problem posed by this group”:^69^

I would like to do a small pilot study and to provide a group of immigrants with flour that has had a supplement of Vitamin D added to it so that they can from this flour make their own chupattees. . . . If in this way we could raise the circulating level of 25 hydroxy-cholecalciferol there would I think be strong grounds for attempting this on a much wider scale. You very kindly said that you would discuss this project with the Panel on Child Nutrition. I hope that they will agree that this is a practical way of approaching the problem.^70^

The “basic science” and analysis underpinning this study, and the harvesting of its biochemical fruits, were done in London, by the Middlesex team, but the pilot study was to be done both geographically and methodologically at a distance: it was to be an intimate, domiciliary clinical study on whole families who were recruited at the Stobhill General Hospital in Glasgow. The clinical work and nutritional observations were to be performed on a deprived—but already well-studied and compliant—urban immigrant population, by experienced local researchers, nutritionists, and public health workers, cooperating with their own local authority but linked to the central government only via O’Riordan’s group. This pattern is obviously reminiscent of Wilson and Surie’s work in colonial India decades before. And as in those early studies, those who were inclined to doubt the conclusions—whether for methodological or political reasons—focused some of their criticism on the necessarily active participation of the study’s clinical material. When the pilot study’s positive results were reported and pressure began to mount on the DHSS and the government to introduce the fortification of chapatti flour, skeptics turned a critical eye on the patients’ self-reported eating habits. For instance, J. A. Sutherland of the Scottish Home and Health Department—a supporter of the idea of fortification in general—wrote with tongue only slightly in cheek to his DHSS colleagues: [Other P-556] “I note that in the trial the chapatti flour was issued free and being Scottish Asians I wonder if they ate more than they would normally have done.”^71^

Policymakers, civil servants, and medical personnel did raise more serious problems with both the O’Riordan study and the use of fortification. Legal problems with the addition of vitamin D to flour, fears of a recurrence of hypercalcemia, concerns that the target populations would not be reached by the fortified staple, especially if it sold at a premium: all contributed to the DHSS’s decision not to mandate the fortification of chapatti flour on a national basis. Of course, these considerations had neither prevented the fortification of margarine during the war, nor led to its removal thereafter. Less reasonably, many experts shared a general conviction that “Asians” would resist fortification on nebulous but “cultural” grounds. As Sylvia Darke of the DHSS bluntly informed her opposite number in the Ministry of Agriculture, Fisheries and Food (MAFF): “if it [chapatti flour] were fortified here we would have the problem of persuading the Asians to use it in the same way that we have difficulty in persuading them to take supplements and get into what sunshine there is!”^72^ H. M. Goodall, in background notes prepared for the response of the head of MAFF to a Parliamentary question in 1976, shared this perception and was additionally wary of racial politics: “Fortification of chappati flour . . . could also cause political, racial, and religious problems because of its specific connection with the Asian community.”^73^ Even this echoes colonial precedents: justifications of governmental inaction in relation to indigenous morbidity and mortality in colonial Africa and India often stressed cultural resistance.^74^ And certainly, such remarks were regarded by members of the ethnic communities, and by local health workers and health authorities, as mere rationalizations of inaction: “many Asian groups are in favour of compulsory fortification; indeed the DHSS has been accused of racism for *not* intervening more actively.”^75^ However, for our present purposes, the Middlesex study’s recapitulation of the idealized colonial model, despite its metropolitan location, is crucially revealing. In the absence of colonies, these elite medical researchers were readily agreeable to colonizing an alternative population: postcolonial immigrants living in medically and economically deprived areas.[Other P-557]

The ethnic communities themselves were not voiceless, and they actively deployed the data and medical attention produced by and about their bodies. In 1976, the Community Relations Committee requested a meeting with the DHSS to address the question of dietary deficiency among the Ugandan Asians. The CRC representatives to the meeting (half of whom were themselves from Asian communities) did accept that “rickets and deficiency states in Ugandan Asian immigrants . . . were primarily due to cultural factors”; nonetheless, they called for the provision of religiously acceptable school meals, and especially “suitable fortification of food.”^76^ K. Nagda, secretary of the Confederation of Indian Organisations, too, sought information and took the DHSS to task for its inaction in 1977; he was fobbed off with the distinctly anodyne reply that the department had published pamphlets for doctors on the subject, and that “discussions are underway with the Health Education council about the issue of health education material in Asian languages, and we have asked them to give priority to leaflets on nutrition.”^77^

Perhaps these ethnic populations could even be said to have colonized their researchers in turn; certainly O’Riordan became a strong advocate of fortification and other active interventions from the DHSS, and opposed its education-alone policy. Writing in 1978 to E. M. Widdowson, head of the Dunn Nutritional Laboratory and then chairing COMA, he complained:

I am rather concerned that so little practical progress seems to have been made in achieving some form of vitamin D fortification to prevent vitamin deficiency in Asian immigrants. I wonder if you could let me know what the current situation is and what the hold up seems to be. It is a long time since we showed that the addition of vitamin D to chaphatti flour, corrected vitamin D deficiency in Asians.^78^

Doctors approaching the problem of “Asian rickets” from a public health or clinical perspective were even more critical. Dr. Sam Tucker, a London pediatrician, argued that while 20 percent of Asians in his district of South-all suffered from rickets, “not one European child” had the condition: “If that’s not a condemnation of our methods, I don’t know what is.”^79^ [Other P-558] Dr. W.T. Cooke of the Birmingham Area Health Authority (and long a thorn in the DHSS flank on the subject of immigrant health) addressed himself to the DHSS’s beleaguered nutrition officer, Sylvia Darke: “You will have seen our paper in the Lancet. Is it not time something was done. . . . I think it is not being very sensible to anticipate changes in dietary habits etc., and general propaganda to effect changes in the situation. Certainly whatever has been put out over the last three years has not been effective!”^80^

By 1979, the left-wing *New Statesman* entered the fray, accusing COMA of being dilatory, and of failing to address “a rickets epidemic” in Asian children, to the great annoyance of the Department.^81^ The liberal *Guardian* newspaper was equally scalding in its criticism, and focused on questions of ethnic prejudice:

Health education workers and minority groups have complained that the DHSS has never publicly acknowledged the seriousness of the rickets problem. . . . Despite frequent representations to the Government, the DHSS has not taken up the suggestion that certain foods eaten by the Asian community, such as chapatti flour, should be fortified with vitamin D. Now Dr. Carlos Ferreyra, chairman of the Community Health Group for Ethnic Minorities, has written to the Prime Minister comparing the present Government’s reaction to rickets to the attitude in the 1940s.

At that time, he said, when rickets was affecting the white population, food was fortified and an extensive programme of nutrition education was undertaken. . . . However, COMA, the DHSS committee looking at aspects of food policy has advised against food fortification and suggested instead only ad-hoc nutritional advice.^82^

Internal memos offer perhaps the best picture of the state of affairs within the DHSS during this crisis. In response to one harshly critical article, Darke wrote to a colleague:

This sort of “article pressure” is always worrying. I think at night of all the Asian children with rickets who could be cured or their illness prevented so easily. Is it my fault? To some extent we have failed. In 1972, our hunch was education but the massive Departmental machine etc etc and our own problems means that [we] cannot easily launch a campaign. What is needed is TV and radio time in short bursts at intervals to repeat and repeat the message. We have not access to radio and TV!! Our pathetic attempt at education via leaflets *is* pathetic. The one action which would *not* solve the problem, and might well land us in [Other P-559] hypercalcaemia is fortification of chupatti flour. . . . It is depressing that a Health and Social Services J. published this stuff. . . . Also, COMA have said *NO FORT* therefore PUBLICITY. HOW DO WE DO IT? TV is the answer.”^83^

Darke was not to get her television ads: the idea was comprehensively dismissed as “a very expensive way of reaching a very small target.”^84^

## Postcolonial Medicine in Britain? Pathologizing British Asians

The use of non-European (or, indeed, impoverished and disempowered European) bodies to advance established research agendas is hardly a novelty in the history of medicine. However, the location of such studies at the heart of elite metropolitan institutions with a general, rather than specialist, remit—university teaching hospitals, not tropical medicine institutes—*is* new, and suggestive of a distinctively “postcolonial medicine” in Britain. The Manchester example hinted at a novel (if still empire-building) integration of clinical, epidemiological, and laboratory research, drawing on local ethnic communities as a research resource; the program at Middlesex reiterated established colonial divisions between basic research and clinical observation. Only the latter came to fruition in the form initially proposed.

In another way, too, U.K. studies of rickets and osteomalacia among Commonwealth immigrants illustrated continuity between colonial medicine and the medical mode that superseded it. Just as colonial medicine stigmatized nonwhite bodies as simultaneously pathological and vulnerable (consider, for example, the model of tuberculosis as a “disease of civilisation”)^85^ and condemned “the diets of native populations” as “defective,” so the prevalence of rickets and osteomalacia among British Asians facilitated critiques both of Asian cultures as pathogenic and of Asian bodies as dangerously unsuited to Britain.^86^ The assumption that the origin of “Asian rickets” was to be sought and found either in culturally specific diet or dress, or in the pigment of Asian skin, pervaded commentaries at every level (sometimes even within groups drawn from the Asian communities themselves, as we have seen above), until the 1980s.[Other P-560]

Assimilation has, for immigrants, long been equated with health, while resistance—often through the preservation of dietary or religious practices—has been similarly linked to illness.^87^ The literature surrounding vitamin D deficiency demonstrates that the cultural choices of Asian immigrants and their families were often conflated with or blamed for physical illness. Thus in a 1963 article entitled “Infantile Rickets Returns to Glasgow,” the authors observed high rates of the disease in recently arrived Asian immigrant families and predicted their swift resolution by means of acculturation alone:

The involvement of Asian children may be due to multiple factors, including habitual consumption of a racial diet, lessened exposure to sunshine, failure of immigrants to learn (or to be taught?) that supplementary intake is essential beneath the smoke pall of a northern city, and the hypothetical possibility that a dusky skin requires more ultraviolet radiation than a white skin. The problem of this racial group is circumscribed and ought to be evanescent. *The solution lies in adequate education of Asian women immigrants firstly to speak English and secondly in the elements of child care in this climate.*^88^

As this quotation suggests, race was considered a contributory factor, echoing the colonial medical literature both in targeting nonwhite racial traits as unhealthy, and in focusing closely on interactions (generally malign) between race and climate among dislocated populations. Other researchers had identified the same “problem”: “It seems a reasonable suggestion that the Pakistani, by virtue of his pigmented skin, requires more sunlight than the native Glaswegian to produce the necessary amount of vitamin D.”^89^

Most articles published during this first wave of “New Commonwealth” immigration focused on the new Asian communities, though often casually likening them to indigenous “problem families.”^90^ “Sunnier lands,”^91^ “dusky skins,” “traditional dress,” and “Asian diets” feature prominently in this medical discourse; British Asians, including those now born and raised in Britain, were likened to “Bedouin women of the Negev.”^92^ By 1965, the *Lancet* was calling for direct observation and supervision of rickets prophylaxis among “young immigrants” by health visitors as a “justifiable extension of preventive medicine in these circumstances.”^93^ The same *Lancet* article used recent studies comparing the vitamin D content of indigenous and immigrant diets in Glasgow (and discovering near-parity) to argue that “skin pigmentation . . . is likely to be the major intrinsic cause” of late rickets among British Asians, even when taking previous dietary habits into account.^94^

Whether or not they accepted such models of racial difference, by the early 1970s researchers across the field were coming to the conclusion that “the combination of dietary habits and social customs among immigrant Asians, including their traditional clothing and their habit of avoiding direct sunlight may be . . . the more important aetiological factors in the production of rickets and osteomalacia.”^95^ In other words, although now construed definitively as a biochemical disease, rickets in Asians was nonetheless assigned a cultural origin.

However, though the clinical literature focused attention on dietary choices and cultural traditions, and on the imperative need for Asians in Britain to assimilate, the demographics of rickets in British Asian communities [Other P-562] could be used to suggest a different and more familiar solution. Even the very earliest studies of “Asian rickets” illustrated the protective impact, particularly for this population (generally impoverished and poorly housed in crowded inner-city areas), of the availability of fortified welfare foods. An influential 1962 study in Glasgow, for example—triggered by the discovery of florid rickets in a fourteen-year-old Pakistani girl—showed that one Asian group was protected from the illness:

They examined 74 adults and children, and found convincing evidence of active rickets or osteomalacia in no less than 35; children between 5 and 15 were most seriously affected. Since all the cases responded rapidly to small doses of calciferol, simple deficiency of vitamin D was apparently responsible. Seeking an explanation for these rather startling observations, the Glasgow workers inquired into the Pakistanis’ dietary habits. Children younger than 5 were receiving welfare foods, which presumably protected them from the disease.^96^

Although both the study and the *Lancet*’s leading article discussing it were much cited in further research, this aspect of its findings received little comment or attention—even from G. C. Arneil and his colleagues, authors of the original work. Similarly, evidence that poor housing (and later, fears of ethnic and racial violence)—rather than modest clothing—might be responsible for elevated levels of osteomalacia and rickets among British Asian women and girls was disregarded.^97^ Whether the medical lack of interest stemmed from a recognition of the intractability of poverty, or from the tractability of rickets to biochemical solutions, is, of course, a matter for speculation. Certainly, the overall response of clinicians and practitioners treating rickets among British Asians was to press for a technical solution—ideally, as the O’Riordan study suggested, the fortification of a basic foodstuff with vitamin D. Chapatti flour, used to make the flatbread that was regarded as a staple of “the Asian diet,” was regarded by many as the ideal vehicle. The ministries, however, enmeshed in free-market policies and cost-cutting, and perhaps overly fearful of potential iatrogenic illness and ethnic discontent with targeted interventions, were deeply opposed.^98^

## The Decline of “Asian Rickets”

By the mid-1970s, then, a new category of rickets had joined “clinical,” “radiological,” and “biochemical” rickets: “Asian” (or “Asiatic”) rickets. Sufferers were sought out and actively treated; however, governmental efforts to *prevent* Asian rickets remained focused not on the provision of attractive supplements, or fortified staple foods, or brighter dwellings, but on educational programs explicitly designed to change culturally sanctioned behavior and dietary choices, and to encourage assimilation. In an unusually direct letter, replying to questions posed by a Glasgow sixth-form student working on a class project, an L. Willcocks of the Department of Health and Social Services summed up the arguments against fortification as they had emerged over the previous decade; the letter is worth citing at length:

Regarding supplementation of food by Vitamin “D,” in 1977, the Committee on Medical Aspects of Food . . . set up a Working Party to report on the need for food fortification with Vitamin D in relation to deficiency disease of all ages in the population. . . . The first decision which had to be made was whether fortification should be “universal” (i.e. applied to all foods of a given type or types) or “selective” (i.e. applied to foods mainly or solely eaten by those at risk). The universal approach was ruled out for a number of reasons, amongst them being that there was a general trend to the view that food should be as “pure” as possible and that there was a segment of the population that has rooted objections to what it sees as “mass medication”—here can be instanced the history of fluoridation in this country. In addition intakes of Vitamin D in excess are known to be harmful and this is a very important factor to be taken into account when the majority of the population in this country do not suffer from a deficiency of the vitamin.

The point about the danger of excess Vitamin D also of course is relevant when considering the case for fortification on a selective basis. Also the eating habits of those from the Indian sub-continent vary considerably.^99^

Willcocks noted that these arguments had turned COMA against additional fortification, and continued with a revealing discussion of the action to be taken instead:

But . . . dietary sources are not the chief or even the most physiological sources of Vitamin D. In fact the chief source is the ultra-violet component of sunlight, and it can be said to be the source with “all the advantages”: it is free, and is natural and being in the open air has many beneficial side effects. It is indeed held by many that the reason why the appearance of rickets has been confined [Other P-564] very largely to girls and women (and to a certain extent children) of Asian origin is that in many cases social customs demands [*sic*] that they be kept in doors and that their clothes should be such as to cover a considerably greater proportion of their body. . . . Thus the natural process of acculturisation [*sic*] might well bring about the complete eradication of rickets. . . . We feel that the best way, in all the circumstances, to deal with the problem is by education . . . to increase the awareness . . . of the problem of rickets and vitamin D deficiency and the relatively simple ways the disease can be combated. As I said, sunlight is free and Vitamin D supplements are available under NHS arrangements.^100^

This assumption that assimilation would cure the “Asian rickets” problem was widespread and almost unexamined. While researchers, health workers, and even policymakers might doubt the efficacy of different strategies aimed at encouraging assimilation, none questioned its benefits—at times comparing British Asians unfavorably to West Indian immigrants, whose health was credited to their more rapid acceptance of “a mode of life more like that of European[s].”^101^

In 1981, researchers still commonly assumed a link between assimilation and health, distinctiveness and disease. One group complained:

Prolonged residence in the United Kingdom and a long period of exposure to western customs was not associated with a better vitamin D status in the adults. The vitamin D status of this community was still markedly inferior to that of a white control group despite attempts to influence their dietary practices and habitual solar exposure.^102^

The authors of another 1981 article, despite noting suggestively that “the absence of European children [in Glasgow] with nutritional rickets since 1975 . . . is attributable to health education and improved living and social conditions,” still attributed rickets among British Asians to “traditional diet” and sun-avoidance rather than a failure to benefit from those improved social conditions.^103^ They argued that “the long-term answer to Asian rickets probably lies in health education and a change towards the [Other P-565] Western diet and lifestyle,” while advocating a practitioner-led drive for increased supplementation in the short term.^104^

Research results challenged many aspects of this model of pathological cultural distinctiveness/curative assimilation. In 1982, a study concluded:

No consistent relationship could be demonstrated between the dietary consumption of vitamin D, phytate, chuppatty flour, fibre, oxalate or meat and the serum 25 hydroxyvitamin D concentration. This supports the view that dietary factors play only a minor role in the aetiology of vitamin D deficiency among Asian immigrants.^105^

But as late as 1986, investigators still felt obligated to test the theory that Asians were racially unsuited, by their darker skins, to the British climate, concluding bluntly that “Indian and Pakistani immigrants have the same capacity as Caucasians to produce vitamin D in response to ultraviolet irradiation.”^106^

By the mid-eighties, the term “Asian rickets” no longer figured in medical indices, and appeared only in the work of a few long-term researchers. Yet through the 1990s, researchers for the Medical Protection Society were still reporting on the dangerous health effects of “Hindu vegetarianism,” and its costs to the NHS. And like these attitudes, rickets also remained in Britain’s Asian communities, despite much-increased outreach and education efforts like Glasgow’s “Stop Rickets” campaign of 1981: “In spite of the extensive medical, social and political attention this condition has received . . . vitamin D deficiency continues to persist in certain Asians in a clinically florid fashion. An effective preventative policy is long overdue.”^107^

## Conclusion

By the late seventies and early eighties, the expert medical community that had developed around “Asian rickets” broadly supported the use of supplementation in one form or another as a short-term response to the problem’s persistence.^108^ Central government persisted with (inexpensive) strategies that centered on education and were at least rooted in, if not explicitly expressive of, assumptions about assimilation. So what does the persistence of vitamin D deficiency among British Asians almost a century after the emergence of simple and cost-effective treatments for the condition reveal about medical approaches to the underlying condition, and to the “ethnic” individuals affected by it?

The case of rickets illustrates the continuation of certain modalities of colonial tropical medicine: the same actors and institutions were involved, and the imperial networks that medicalized culture and race in the former colonies and the New Commonwealth came into play equally in conceptualizing immigrant and ethnic minority health needs at home. Sociologists (and a few historians) have already pointed out that attention to the medical needs of Asian, African-Caribbean, Cypriot, and other minority ethnic groups has come primarily from clinicians and health policymakers—and thus only indirectly reflects those communities’ own perceptions and medical experiences.^109^ Clinicians and policymakers have focused on the novel, the “interesting,” and the “tropical” aspects of health and disease in these emergent communities, perhaps to the exclusion of the mundane, the commonplace, and the metropolitan. They have also focused on aspects that fit well with existing or emerging research agendas. Thus, comparatively greater space has been given in the medical press and the policy agenda to rickets (and the adult condition of osteomalacia), tuberculosis, and sickle-cell anemia among different ethnic groups, than to diabetes, asthma, or lack of access. More [Other P-567] specifically, medical and policy responses to rickets and osteomalacia among British Asians demonstrated the ways in which clinical attention to diet (as an example of the intersection between culture and biology) was directive and normative: it again and again argued for the benefits of assimilation, and the dangers of maintaining particular, distinctive, cultural practices. And the case of rickets also suggests that postcolonial governmental responses, like those of the preceding empire, were often shaped as much by financial as by medical considerations, especially when only “ethnic” populations were at risk.

Asian immigrants and their doctors were given two choices: rickets and osteomalacia could be “Asian”—in other words, caused by the dietary and dress choices of Pakistani, Bangladeshi, and Indian immigrants and their families—or they could be “English”: caused by the unfortunate climate and northern latitude of the United Kingdom (sometimes in combination with the much-discussed “dusky skin” of these communities). But either way, in the eyes of medical policymakers, the disease could—and therefore should—be cured by assimilative behavioral changes: changes to make British Asians less Asian or more English. And because assimilation could become cure, responsibility for the two diseases was pinned firmly on the affected communities, families, and individuals themselves. This was a clear shift from British policy responses to rickets in the indigenous community before, and especially during, World War II. And it is far from coincidental that this medical discourse on the malign impacts of “alien” culture on individual bodies ran in parallel to a political discourse predicting similarly disastrous results from the interpenetration of the British body politic by “alien” cultural elements. If, in the end, there were no rivers of blood, there were certainly no rivers of (vitamin D– fortified) milk, either.[Other P-568]

## Footnotes

1
	  The following sample the different approaches to and contexts of colonial and imperial medicine: WarwickAnderson“Immunities of Empire: Race, Disease, and the New Tropical Medicine, 1900–1920,”Bull. Hist. Med.19967094118885007210.1353/bhm.1996.0002 DavidArnoldColonizing the Body: State Medicine and Epidemic Disease in Nineteenth-Century IndiaBerkeleyUniversity of California Press1993 AmyFairchildScience at the Borders: Immigrant Medical Inspection and the Shaping of the Modern Industrial WorkforceBaltimoreJohns Hopkins University Press2003 MarkHarrisonMichaelWorboys“A Disease of Civilisation: Tuberculosis in Britain, Africa and India, 1900–1939,”Migrants, Minorities and Health: Historical and Contemporary StudiesLaraMarksMichaelWorboysLondonRoutledge199793124 MarkHarrisonPublic Health in British India: Anglo-Indian Preventive Medicine, 1859–1914CambridgeCambridge University Press1994 DanielHeadrickTools of Empire: Technology and European Imperialism in the Nineteenth CenturyOxfordOxford University Press1981 ShulaMarks“What Is Colonial about Colonial Medicine? And What Has Happened to Imperialism and Health?”Soc. Hist. Med.199710205191161949310.1093/shm/10.2.205 MeganVaughanCuring Their Ills: Colonial Power and African IllnessOxfordPolity Press1991 LuiseWhiteSpeaking with Vampires: Rumor and History in Colonial AfricaBerkeleyUniversity of California Press2000 MichaelWorboys“The Colonial World as Mission and Mandate: Leprosy and Empire, 1900–1940,”Osiris2001152071810.1086/64932711973829 Worboys“Tuberculosis and Race in Britain and Its Empire, 1900–50,”Race, Science and Medicine, 1700–1960WaltraudErnstBernardHarrisLondonRoutledge199914466 . For critiques of this literature, see WarwickAnderson“Where Is the Postcolonial History of Medicine?”Bull. Hist. Med.19987252230978045110.1353/bhm.1998.0158 Anderson“How’s the Empire? An Essay Review,”J. Hist. Med. & Allied Sci.20035845965 . See also RandallPackard“Postcolonial Post-Colonial Medicine,”Medicine in the Twentieth CenturyRogerCooterJohnPickstoneAmsterdamHarwood Academic Press200097112
	  
	

2
	  The actualities of colonial medicine have recently been explored from a range of perspectives, particularly in relation to the input of indigenous practitioners, and forms of popular resistance. See, e.g., HeatherBellFrontiers of Medicine in the Anglo-Egyptian Sudan, 1899–1940OxfordOxford University Press1999 Bell“Midwifery Training and Female Circumcision in the Inter-War Anglo-Egyptian Sudan,”J. Afr. Hist.199839293312 ChristianHochmuth“Patterns of Medical Culture in Colonial Bengal, 1835–1880,”Bull. Hist. Med.20068039721654988110.1353/bhm.2006.0021 SarahHodges“‘Looting’ the Lock Hospital in Colonial Madras during the Famine Years of the 1870s,”Soc. Hist. Med.20051837998
	  
	

3
	  See, e.g., ColinHolmesJohn Bull’s Island: Immigration and British Society, 1871–1971LondonMacmillan1988), esp. chap. 5 (pp. 20972
	  
	

4
	  As a starting point, see HolmesJohn Bull’s Island (n. 3) ColinHolmesA Tolerant Country? Immigrants, Refugees, and Minorities in BritainLondonFaber and Faber1991 DanielLawrenceBlack Migrants, White Natives: A Study of Race Relations in NottinghamCambridgeCambridge University Press1974 PanikosPanayiRacial Violence in Britain in the Nineteenth and Twentieth Centuriesrev. ed.LondonLeicester University Press1996 KathleenPaulWhitewashing Britain: Race and Citizenship in the Postwar EraIthacaCornell University Press1997 RobertWinderBloody Foreigners: The Story of Immigration to BritainLondonAbacus Press2004esp. chaps. 20–22
	

5
	For examples of the twentieth-century relationship between medicine and immigration, see FairchildScience at the Borders (n. 1) AlanKrautSilent Travelers: Germs, Genes, and the “Immigrant Menace”BaltimoreJohns Hopkins University Press1995 NayanShahContagious Divides: Epidemics and Race in San Francisco’s ChinatownBerkeley and Los AngelesUniversity of California Press200112395801 . Little has yet been published on this subject in the United Kingdom, but see KristaMaglen“Intercepting Infection: Quarantine, the Port Sanitary Authority, and Immigration in Late Nineteenth and Early Twentieth Century Britain”Ph.D. diss.University of Glasgow2001 MarksWorboysMigrants, Minorities (n. 1)

6
    In ongoing research, I am examining the perspectives of individual immigrant and British Asian patients, their families, and their communities. Here, however, I am primarily concerned with medical and policy responses to these groups. In the category of “visible responses” I include such material as research papers, editorials, letters, grant applications, and policy-statements from professional organizations.

7
    No responsible or rigorous historian of medicine could argue that medical researchers, colonial or postcolonial, were unconcerned about the health or medical needs of their patients. Those studying rickets in Britain’s Asian communities were certainly motivated to treat, as well as to study, the phenomena of vitamin D deficiency; some became advocates for their patient-communities. However, in general, their interest in the condition stemmed from, and their approaches to it were driven by, its scientific rather than its humanitarian merit.

8
    See MelbourneTapper“An ‘Anthropathology’ of the ‘American Negro’: Anthropology, Genetics, and the New Racial Science, 1940–1952,”Soc. Hist. Med.19971026389 , for another perspective on this phenomenon, in the case of sickle-cell anemia. 1161949610.1093/shm/10.2.263

9
    The language of “colonization” is that of the actors themselves. For instance, John Fishwick, town clerk of the London borough of Lambeth, complained to the Ministry of Health in February 1955: “A complication is that coloured immigrants naturally gravitate on first arrival to ‘colonies’ of their kind and remain there” (The National Archives, Public Record Office, Kew, Richmond, Surrey [TNA/PRO], MH 58/670). On the colonial case, see MichaelWorboys“The Emergence of Tropical Medicine: A Study in the Establishment of a Scientific Specialty,”Perspectives on the Emergence of Scientific DisciplinesGerardLemaineRoyMacLeodMichaelMulkayPeterWeingartChicagoAldine Press19767396 , on p. 83 : “The investigation and teaching of the etiology and treatment of tropical diseases was developing in an environment and culture totally different from the tropics. Work on etiology became exclusively scientific, based on parasitological studies and the germ theory of disease. The clinical treatment of these diseases took precedence over prevention and epidemiological studies of disease incidence and control. In the metropolitan situation, remote from the practice problems of the tropics, the study of tropical diseases became increasingly preoccupied with scientific problems rather than with the problems of poor health.”

10
    TNA/PRO FD 1/1974. The Medical Research Committee (later Council) was established in 1913 to advise the government on matters of medical research relating to tuberculosis, and to distribute funds. Fletcher’s skillful navigation and exploitation of wartime circumstances, however, saw the nascent organization transformed from a narrowly focused, tightly controlled, disease-specific research committee to a broadly defined, ambitious research organization with a great deal of autonomy in its own affairs; see JoanAustoker“Walter Morley Fletcher and the Origins of a Basic Biomedical Research Policy,”Historical Perspectives on the Role of the MRC: Essays in the History of the Medical Research Council of the United Kingdom and Its Predecessor, the Medical Research Committee, 1913–1953AustokerLindaBryderOxfordOxford University Press19892333 . The Ministry of Health, on the other hand, emerged as part of government in 1919, from a wartime promise made by Prime Minister David Lloyd George for a healthier Britain, fit for returning heroes; see DorothyPorterHealth, Civilization and the State: A History of Public Health from Ancient to Modern TimesLondonRoutledge1999 The two organizations drew up a concordat in 1924 to delineate research “territory,” with the Ministry having responsibility for “applied” research, while the MRC took responsibility for directing and funding “new” research. Although the terms of the document have changed over the years, a similar agreement survives to this day.

11
    See, e.g., ManeeshaLal“The Politics of Gender and Medicine in Colonial India: The Countess of Dufferin’s Fund, 1885–1888,”Bull. Hist. Med.19946829668173304 GeraldineForbes“Medical Careers and Health Care for Indian Women: Patterns of Control,”Women’s Hist. Rev.1994351530

12
    Walter Fletcher to A. V. Hill, 29 January 1929, TNA/PRO FD 1/1974.

13
    Dagmar CurgelWilsonEllaSurie“Osteomalacia (Late Rickets) Studies. III. Dietary Factors in the Aetiology of Osteomalacia,”1929214 TNA/PRO FD 1/1974

14
    Ibid., p. 4.

15
    DanielsC. W.“Filariae and Filarial Disease in British Guiana,”J. Trop. Med.1898115 , cited in Anderson“Immunities of Empire” (n. 1) 113

16
    See Anderson“Immunities of Empire” (n. 1) ArnoldColonizing the Body (n. 1). Of course, an interest in habits and customs also typified particularly sanitarian studies of the European and American “underclass,” and many studies of African American populations.

17
    Walter Fletcher to George Newman, 13 November 1929, TNA/PRO FD 1/1974 (emphasis added). The letter also reflected the strong bias of the MRC toward biochemical models and biomedical scientists, rather than clinicians as researchers.

18
    See ArnoldColonizing the Body (n. 1), for the archetypal examples of variolation and pilgrimage practices in colonial India, and for the more cautious approach taken later in relation to plague. And see Bell“Midwifery Training” (n. 2) , for a counterexample.

19
    WilsonSurie“Osteomalacia” (n. 13) 14

20
    See Kathleen OlgaVaughanThe Purdah System and Its Effect on Motherhood: Osteomalacia Caused by the Absence of Light in IndiaCambridgeHeffer1928 Such research results might or might not be published monographically or in journals dedicated to colonial or tropical medicine; however, these publications were little cited by policymakers addressing British medicine or medical needs. I have found no reference to sources published in Indian or Pakistani journals, or by authors with identifiably Indian or Pakistani names, in the official records for this period, save for a few collaborations (generally based in British teaching hospitals) with recognized British researchers.

21
    See CeliaPetty“Primary Research and Public Health: The Prioritization of Nutrition Research in Inter-war Britain,” in Austoker and BryderHistorical Perspectives (n. 10) 83108 DavidF. SmithNutrition in Britain: Science, Scientists and Politics in the Twentieth CenturyLondonRoutledge1997 HarmkeKammingaAndrewCunninghamThe Science and Culture of Nutrition, 1840–1940AmsterdamRodopi1995

22
    RimaAppleVitamania: Vitamins in American CultureRutgersRutgers University Press19963353 For a timeline of research on vitamin D, see RobertaConlanElizabethShermanUnraveling the Enigma of Vitamin DWashington, D.C.National Academy of Science2000 Web-published at www.beyonddiscovery.org accessed 12 December 2004

23
    SmithDavid F.MalcolmNicholson“Chemical Physiology Versus Biochemistry, the Clinic Versus the Laboratory: The Glaswegian Opposition to Edward Mellanby’s Theory of Rickets,”Proc. Roy. Coll. Physicians Edinburgh198919516011612466

24
    WilfredSheldon“Observations on Rickets,”Lancet1935225134

25
    Craniotabes could be distinguished by the distinctive “rubber ball” (and later, “eggshell crackling”) of sensations it presented to the experienced touch on an affected infant’s skull.

26
    “Rickets,”Lancet1940235848584

27
    See SmithNicholson“Chemical Physiology” (n. 23) 5559

28
    AnneMurcott“Food and Nutrition in Post-War Britain,”Understanding Post-War British SocietyJamesObelkevichPeterCatterallLondonRoutledge199415564

29
    DavidKrestin“Treatment of Rickets with Single Massive Doses of Vitamin D2,”Lancet194524578183 TNA/PRO MH 55/1558, MH 55/2336, and MAF 256/219 also document the emergence of an increasingly judgmental stance.

30
    See CharlesWebster“Government Policy on School Meals and Welfare Foods, 1939–1970,” in SmithNutrition in Britain (n. 21) 190213 Ministry of Health and Department of Health for ScotlandReport of the Joint Sub-Committee on Welfare FoodsLondonHMSO1957 W. T. C.Berry“Nutritional Aspects of Food Policy,”Proc. Nutr. Soc.19682718569754010.1079/pns19680003

31
    The papers of Charles E. Dent, a pioneer in the field of metabolic rickets and founder of the University College Hospital Metabolic Ward in 1951, offer invaluable material on this subject: Contemporary Medical Archives Centre, Wellcome Library, London (CMAC), PP/CED, esp. PP/CED/C.3/1/2, PP/CED/C.3/1/3, PP/CED/E.1/65, and PP/CED/E.1/77 (the first two files illustrate collaborations with Glaxo and Bayer; the last two, Dent’s view of the shift from nutritional to metabolic rickets).

32
    Webster“Government Policy” (n. 30)

33
    “Draft letter to Hospitals/Consultants from Secretary, Vitamin D subcommittee of the BPA, 5 January 1959,” TNA/PRO MH 55/2335.

34
    Medical Research Council, “Report of the Conference on Hypercalcaemia in Infants,” May 1956, p. 1, TNA/PRO MAF 256/219. By 1971, the Food and Agriculture Organization and the World Health Organization were using Britain as a model of the risks of oversupplementation with a potentially toxic nutrient: FAO/WHO, *Joint FAO/WHO Expert Committee on Nutrition, Eighth Report: Food Fortification, Protein-Calorie Malnutrition*, World Health Organization Technical Report Series, no. 447 (1971), pp. 27–28.

35
    StewartW. K.MitchellR. G.MorganH. G.LoweK. G.ThomsonJ.“The Changing Incidence of Rickets and Hypercalcaemia as Seen in Dundee,”Lancet196428367982 on p. 680 1410795410.1016/s0140-6736(64)91516-8

36
    The files of the British Ministries of Health (MoH) and Agriculture, Fisheries and Food (MAFF) and the Division of Maternal and Child Welfare offer rich evidence of these debates and the intersections between food manufacturers, food policy, and concerns about “mass medication” and the public health. See, e.g., TNA/PRO MH 55/2335, MH 55/2336, MAF 256/219. Some historical analysis of these debates is provided in Petty, “Primary Research” (n. 21); and see, of course, *Hansard* for both the House of Commons and House of Lords.

37
    MRC, “Report of the Conference on Hypercalcaemia in Infants” (n. 34), p. 2.

38
    In the 1940s margarine was selected as the vehicle for mandatory vitamin D supplementation, along with the National Dried Milk, based on the dietary habits of the majority population, in combination with the rationing of butter.

39
    PQ 1498/72/73, 14 March 1973, *Parliamentary Debates*, Lords, 5th ser., vol. 340, cols. 300–303.

40
    For examples and cases, see MacLeodRoyLewisMiltonDisease, Medicine and Empire: Perspectives on Western Medicine and the Experience of European ExpansionLondonRoutledge1988 PatiBiswamoyHarrisonMarkHealth, Medicine and Empire: Perspectives on Colonial IndiaHyderabadOrient Longman2001 Tapper“‘Anthropathology’” (n. 8); Michael Worboys, “Germs, Malaria and the Invention of Mansonian Tropical Medicine: From ‘Diseases in the Tropics’ to ‘Tropical Diseases,’”Warm Climates and Western Medicine: The Emergence of Tropical Medicine, 1500–1900ArnoldDavidAmsterdamRodopi19961812078783626 BeinartJennifer“The Inner World of Imperial Sickness: The MRC and Research in Tropical Medicine,” in Austoker and BryderHistorical Perspectives (n. 10) 10935

41
    On the programmatic aims and methods of tropical medicine, see David Arnold, “Introduction: Tropical Medicine before Manson,” in Arnold, *Warm Climates* (n. 40), pp. 1–19; Worboys, “Germs, Malaria” (n. 40). On nutritional diseases as tropical medicine, see Beinart, “Inner World” (n. 40). Not all fieldwork was done in tropical locations: the working-class slums of Britain and Europe were also research sites (in nutritional work, particularly after World War I). Their populations were “colonized” by biomedicine in almost identical ways—a fact that either problematizes or valuably extends the concept of “colonial medicine,” depending on the interpreter’s historiographic perspective. But see LevinePhilippaProstitution, Race, and Politics: Policing Venereal Disease in the British EmpireNew YorkRoutledge2003 , for a cogent discussion of differences between even ostensibly similar practices in colonial and metropolitan settings.

42
    See, e.g., SheihamHelenQuickAllisonThe Rickets Report: Why Do British Asians Get Rickets?LondonHaringey Community Health Council19826

43
    See ArneilG. C.CrosbieJ. C.“Infantile Rickets Returns to Glasgow,”Lancet1963282423251404727310.1016/s0140-6736(63)92170-6 ; Panel on Child Nutrition: Meeting of the Chairmen of Working Groups, 18 December 1972, p. 1, TNA/PRO MH 148/623; and below.

44
    E.g., in chronological order, FordJ. A.“Proceedings: Aetiology of Asian Rickets and Osteomalacia in the United Kingdom,”Arch. Dis. Childhood19734882728474969810.1136/adc.48.10.827-bPMC1648529 Ford“Asian Rickets and Osteomalacia,”Nursing Times19747049504813622 StanburyS. W.TorkingtonP.LumbG. A.AdamsP. H.de SilvaP.TaylorC. M.“Asian Rickets and Osteomalacia: Patterns of Parathyroid Response in Vitamin D Deficiency,”Proc. Nutr. Soc.19753411117110315410.1079/pns19750021 RobertsonI.KelmanA.DunniganM. G.“Chapatty Intake, Vitamin D Status and Asian Rickets,”Brit. Med. J.197712293083208810.1136/bmj.1.6055.229-dPMC1604393 FordJ. A.McIntoshW. B.HaaseS.WrightA. W.DunniganM. G.“Treatment of Severe Asian Rickets with Vitamin D-Fortified Chupatti Flour,”Arch. Dis. Childhood1977527434492132910.1136/adc.52.9.743-aPMC1544748 DunniganM. G.RobertsonI.“Residence in Britain as a Risk Factor for Asian Rickets and Osteomalacia,”Lancet1980315770610318610.1016/s0140-6736(80)91265-9 DunniganM. G.McIntoshW. B.SutherlandG. R.“Policy for Prevention of Asian Rickets in Britain: A Preliminary Assessment of the Glasgow Rickets Campaign,”Brit. Med. J. (Clin. Res. ed.)19812823576010.1136/bmj.282.6261.357PMC15041956780022 “Asian Rickets in Britain,”Lancet19813184026115164 As the list of authors suggests, this is a small community—but a prolific and highly influential one. I found no evidence of reservations about this terminology, which was widely used within articles and policy papers.

45
    Berry, “Nutritional Aspects” (n. 30), p. 3.

46
    Ibid., p. 7.

47
    Ibid., p. 3.

48
    COMA Panel on Child Nutrition, “Minutes of the meeting held on October 5 1972 at the DHSS,” CMAC PP/CED/B.2/5.

49
    ValierHelen K.“The Politics of Scientific Medicine in Manchester, c. 1900–1960” D.Phil. diss. University of Manchester2002 ValierHelen K.PickstoneJohn V.The Manchester Royal Infirmary, 1945–2002 in press 108

50
    Quoted in COMA, *Interim Report on Vitamin D by the Panel on Child Nutrition; First Report of the Panel on Nutrition of the Elderly* (London: Stationery Office, 1970), pp. 12–13. See also TNA/PRO MH 55/2335 for records of this study.

51
    C. E. Dent to J. Kyle Smith, 10 September 1960, CMAC PP/CED/C.3/1/3.

52
    Among some workers, low rates of referral had become a cause for despair by 1972. See DawsonK. P.MondheM. S.“Nutritional Rickets among the Immigrant Population of Bradford,”Practitioner197220878991 on p. 790 5044966 : “Despite the publicity given to the patterns of illness in immigrants, we have been surprised by the complete absence of reference to rickets or vitamin D deficiency in the referral letters from general practitioners.”

53
    Dent to Dr. Tizard, 14 July 1956, CMAC PP/CED/C.3/1/3.

54
    Although this was and is the most common way in which any/all patients can be at the heart of research, it is not the only way: see ValierHelenBivinsRoberta“Organization, Ethnicity and the British National Health Service,”Innovations in Health and Medicine: Diffusion and Resistance in the Twentieth CenturyStantonJenniferLondonRoutledge20023764 , for an alternative model of patient and community involvement, as displayed in Manchester’s responses to sickle-cell anemia and thalassemia.

55
    On colonial malnutrition, see Beinart“Inner World” (n. 40) 12126 WorboysMichael“The Discovery of Colonial Malnutrition between the Wars,”Imperial Medicine and Indigenous SocietiesArnoldDavidManchesterManchester University Press198820825 . For the notion of (racially or regionally identified) disease as a commodity, see WailooKeithDying in the City of the Blues: Sickle Cell Anemia and the Politics of Race and HealthChapel HillUniversity of North Carolina Press2001

56
    Glasgow research, of course, drew upon a heritage of environmental rather than molecular explanations of rickets, while research output from Birmingham and Bradford focused strongly on public health and population studies of the reemergent disease.

57
    See, e.g., Sheiham and Quick, *Rickets Report* (n. 42); Community Nutrition Group, “Food and Ethnic Minorities,” Information Sheet 16, 1989; Health Education Authority, “Nutrition in Minority Ethnic Groups,” 1991. The Glasgow researchers studying phytate and promoting fortification did distinguish between different “Asian” populations (albeit with some prodding from Subcontinental colleagues—e.g., TeotiaS. P. S.TeotiaM.“Nutritional Rickets in Immigrants,”Brit. Med. J.1972411112507744810.1136/bmj.4.5832.111-cPMC1786209 : see, e.g., PietrekJ.PreeceM. A.WindoJ.O’RiordanJ. L. H.“Prevention of Vitamin-D Deficiency in Asians,”Lancet19763071145485819710.1016/s0140-6736(76)91539-7

58
    COMA Panel on Child Nutrition, “Minutes of the meeting held on October 5 1972” (n. 48).

59
    Panel on Child Nutrition: Meeting of the Chairmen (n. 43), p. 1.

60
    For figures on tuberculosis morbidity and mortality among immigrants, related debates, and examples of public and press sentiments on the subject, see TNA/PRO MH 55/2275 and 55/2276.

61
    S. W. Stanbury to J. M. L. Stephen, 6 March 1973, p. 1 (emphasis added), TNA/PRO MH 148/623.

62
    Ibid., p. 2.

63
    Ibid., pp. 2–3.

64
    See C. E. Dent and J. O’Riordan, “Project for Joint UCH/Middlesex Hospital Research on Metabolic Bone Disease,” 20 March 1974, CMAC PP/CED/A.2/2.

65
    See, e.g., PreeceM. A.FordJ. A.McIntoshW. B.DunniganM. G.TomlinsonS.O’RiordanJ. L. H.“Vitamin D Deficiency among Asian Immigrants to Britain,”Lancet19733019071010.1016/s0140-6736(73)91361-54123837

66
    Ibid., p. 910.

67
    Stephen Tomlinson, interviewed by author, 22 April 2004, Heath Hospital, Cardiff, Wales. This is not to say that they were not interested in finding a solution for their patients and the British Asian community—indeed, members of the group became active supporters of fortification programs. See, e.g., Pietrek et al., “Prevention” (n. 57), and other group publications post-1974.

68
    J. L. H. O’Riordan to J. M. L. Stephen, 22 May 1973, TNA/PRO MH 148/623.

69
    P. J. Everett [Committee for Community Medicine, British Medical Association] to R. P. Pole [Public and Environmental Health Division, DHSS], 16 June 1978, TNA/PRO MH 148/624.

70
    O’Riordan to Stephen, 22 May 1973, TNA/PRO MH 148/623 (a chapatti is an unleavened flatbread made of a mixture of wholegrain and other wheat flour).

71
    J. A. Sutherland to L. G. Smith, 5 September 1975, ibid.

72
    S. J. Darke to Dr. G. A. H. Elton, 13 February 1975, ibid.

73
    H. M. Goodall, “Background notes and draft answer,” 7 May 1976, ibid.

74
    The classic studies of this phenomenon in the literature are Arnold, *Colonizing the Body*, and Vaughan, *Curing Their Ills* (both n. 1). For earlier examples in relation to nonwhite immigration, see Nayan Shah, *Contagious Divides* (n. 5).

75
    SheihamQuickRickets Report (n. 42) 24 (emphasis in original)

76
    “Health Education and Diet for Ugandan Asians: Meeting with Community Relations Commission 19 October, 1976,” p. 1, TNA/PRO MH 148/624.

77
    S. J. Darke to K. Nagda, 22 December 1977, ibid.

78
    O’Riordan to E. M. Widdowson [Chairman of COMA, Dunn Nutritional Laboratory], 31 July 1978, ibid.

79
    Quoted in Derek Humphrey, “MP Seeks to Curb Rickets,” *Sunday Times*, 20 November 1977, clipping in TNA/PRO MH 148/624.

80
    Dr. W. T. Cooke to S. J. Darke, 5 May 1977, TNA/PRO MH 148/624.

81
    “Rickets in Asian Communities,” *New Statesman*, 23 February 1979, clipping in TNA/PRO MH 148/624; D. K. Smith to L. Fosh, 28 February 1979, TNA/PRO MH 148/624.

82
    “Aid Urged for Asians Affected by Rickets,” *Guardian*, 5 July 1979, clipping in TNA/PRO MH 148/624.

83
    Memo, Darke to Dr. A. Yarrow, 13 December 1978 (emphases in original), TNA/PRO MH 148/624.

84
    Memo, Yarrow to Darke, 21 December 1978, ibid.

85
    Harrison and Worboys, “Disease of Civilization” (n. 1).

86
    MRC, *Annual Report, 1937–38* (London: HMSO, 1939), p. 17, quoted in Beinart, “Inner World” (n. 40), p. 125.

87
    There have, however, been exceptions to the rule: clinicians have often noted certain areas in which difference has been associated with health. For example, in the first half of the twentieth century, medical professionals frequently cited the diets of Jewish and East Asian immigrants in explaining the health of those supposedly degenerate populations. See Lara Marks and Lisa Hilder, “Ethnic Advantage: Infant Survival among Jewish and Bengali Immigrants in East London, 1870–1990,” in MarksWorboysMigrants, Minorities (n. 1) 179209

88
    Arneil and Crosbie, “Infantile Rickets Returns” (n. 43), on p. 424.

89
    “Rickets and Osteomalacia,”Lancet1962279116869 on p. 1168

90
    See, e.g., StewartW. K.MitchellR. G.MorganH. G.LoweK. G.ThomsonJ.“The Changing Incidence of Rickets and Infantile Hypercalcaemia As Seen in Dundee,”Lancet196428367982 p. 679 1410795410.1016/s0140-6736(64)91516-8 : “Recently a surprisingly high incidence of rickets has been reported in the Pakistani community in Glasgow . . . and in various immigrant communities (mostly coloured), in London. . . . Except in such communities and in problem families, nutritional rickets is no longer a major problem.”

91
    Ibid., p. 681.

92
    WillsM. R.PhillipsJ. B.DayR. C.BatemanE. C.“Phytic Acid and Nutritional Rickets in Immigrants,”Lancet197229977173 p. 772 411124810.1016/s0140-6736(72)90523-5

93
    “Annotations: Rickets in Immigrant Children,”Lancet196528511067 on p. 1107 14283774

94
    Ibid.

95
    DentC. E.RoweD. J. F.RoundJ. M.StampT. C. B.“Effect of Chapattis and Ultraviolet Irradiation on Nutritional Rickets in an Indian Immigrant,”Lancet1973301128284 p. 1284 412607510.1016/s0140-6736(73)91296-8 . See also Wills“Phytic Acid” (n. 92) HodgkinP.KayG. H.HineP. M.LumbG. A.StanburyS. W.“Vitamin-D Deficiency in Asians at Home and in Britain,”Lancet197330216773 412425010.1016/s0140-6736(73)93004-3 (with its piquant assumption that “Home” for Asians was in South Asia); GoelK. M.SweetE. M.LoganR. W.“Florid and Subclinical Rickets among Immigrant Children in Glasgow,”Lancet1976307114145 (and *Lancet* passim) 10.1016/s0140-6736(76)91538-558196 StampT. C. B.“Factors in Human Vitamin-D Nutrition, and in the Production and Cure of Classical Rickets,”Proc. Nutr. Soc.19753411930 (and *Proc. Nutr. Soc.* passim) 17167410.1079/pns19750022 FordJ. A.ColhounE. M.McIntoshW. B.DunniganM. G.“Rickets and Osteomalacia in the Glasgow Pakistani Community, 1961–1971,”Brit. Med. J.1972267780503170910.1136/bmj.2.5815.677PMC1788872 Barbara MawerE.HolmesAnne M.“Rickets in Glasgow Pakistanis,”ibid.317778 (and *Brit. Med. J.* passim) 10.1136/bmj.3.5819.177-cPMC17885894339114 . Meanwhile, Stanbury’s bristling response to press assumptions of racial (e.g., skin pigmentation) etiology for “Asian rickets” in interpreting his group’s research results reveals the persistence of that claim in popular culture: see StanburyS. W.“Vitamin-D Deficiency in Asians,”Lancet1973302446412492710.1016/s0140-6736(73)92311-8

96
    “Rickets and Osteomalacia” (n. 89), p. 1168.

97
    For a discussion of such factors and fears, see Sheiham and Quick, *Rickets Report* (n. 42), pp. 35–36. Not all medical professionals ignored the role of poor-quality housing and hostile social conditions: see GertnerJ. M.LawrieBrenda“Preventing Nutritional Rickets,”Lancet19773092576478610.1016/s0140-6736(77)91056-x

98
    See TNA/PRO MH 148/264.

99
    L. Willcocks to Caroline Ralston, 17 March 1980, p. 1, ibid.

100
    Ibid., p. 2.

101
    StephensJoan“Epidemiological and Dietary Aspects of Rickets and Osteomalacia,”Proc. Nutr. Soc.19753413175 on p. 137 . For other examples, see Dunnigan and Robertson, “Residence in Britain as a Risk Factor” (n. 44); Goel et al., “Florid and Subclinical Rickets” (n. 95); Pietrek et al., “Prevention” (n. 57); Wills et al., “Phytic Acid” (n. 92). 110315510.1079/pns19750023

102
    StephensW. P.KlimiukP. S.WarringtonS.TaylorJ. L.BerryJ. L.MawerE. B.“Observations on the Natural History of Vitamin D Deficiency amongst Asian Immigrants,”Quart. J. Med.19825117188 on p. 171 6981122

103
    GoelK. M.SweetE. M.CampbellS.“Reduced Prevalence of Rickets in Asian Children in Glasgow,”Lancet19813184057 on p. 406 611516810.1016/s0140-6736(81)90842-4

104
    Ibid., p. 405.

105
    StephensW. P.KlimiukP. S.SWarringtonTaylorJ. L.“Observations on the Dietary Practices of Asians in the United Kingdom,”Hum. Nutr.—Appl. Nutr.19823643844 on p. 444 7161136

106
    LoC. W.ParisP. W.HolickM. F.“Indian and Pakistani Immigrants Have the Same Capacity as Caucasians to Produce Vitamin D in Response to Ultraviolet Irradiation,”Amer. J. Clin. Nutr.19864468385 on p. 683 376645410.1093/ajcn/44.5.683

107
    IqbalS. J.KaddamI.WassifW.NicholF.WallsJ.“Continuing Clinically Severe Vitamin D Deficiency in Asians in the UK (Leicester),”Postgrad. Med. J.19947070814 on p. 708 783116510.1136/pgmj.70.828.708PMC2397799 . See also LawsonM.ThomasM.“Vitamin D Concentrations in Asian Children Aged 2 Years Living in England: Population Survey,”Brit. Med. J.199931828987287910.1136/bmj.318.7175.28PMC27672 WhartonBrianBishopNick“Rickets,”Lancet20033621389140010.1016/S0140-6736(03)14636-314585642

108
    Many of the articles cited above, published from the mid-1970s onward, advocate supplementation; see also the influential editorial in the *Lancet*, “Asian Rickets in Britain” (n. 44), which lambasted “ineffective preventive action by health services” and condemned governmental resistance to supplementation as a “pursuit of perfection [that] may remain the enemy of progress” (p. 402).

109
    See, e.g., DonovanJenny“Ethnicity and Health: A Research Review,”Soc. Sci. & Med.19841966370639069110.1016/0277-9536(84)90237-5 Donovan“We Don’t Buy Sickness, It Just Comes”: Health, Illness and Health Care in the Lives of Black People in LondonAldershotGower1986 KelleherD.HillierS.Researching Cultural Differences in HealthLondonRoutledge1996 ShahNayanContagious Divides (n. 5) ValierBivins“Organization, Ethnicity” (n. 54) WailooDying in the City of the Blues (n. 55) RamakrishnaJ.WeissM.“Health, Illness and Immigration: East Indians in the United States,”Western J. Med.199215726570PMC10112741413767

